# Screen Printing Conductive Inks on Textiles: Impact of Plasma Treatment †

**DOI:** 10.3390/s25134240

**Published:** 2025-07-07

**Authors:** Julia Guérineau, Jollan Ton, Mariia Zhuldybina

**Affiliations:** 1 Systems Engineering Department, École de Technologie Supérieure, Montréal, QC H3C 1K3, Canada; julia.guerineau@etsmtl.ca; 2 Electrical Engineering Department, École de Technologie Supérieure, Montréal, QC H3C 1K3, Canada; jollan.ton.1@ens.etsmtl.ca

**Keywords:** e-textiles, smart textiles, flexible electronics, screen printing, conductive inks, plasma treatment, wearable technology, Internet of Things

## Abstract

**Highlights:**

**What are the main findings?**

- Plasma treatment is shown to play a role in optimizing the electrical properties of printed silver-based inks.
- Applying plasma treatment tends to increase the thickness of the printed ink and concomitantly decrease sheet resistance.

**What are the implications of the main findings?**

- Combining nitrogen cleaning and plasma treatment shows promise for improving ink transfer properties and ink–textile contact, which may improve washability.
- Plasma treatment offers a promising research avenue for enhancing encapsulation or serving as a substitute for it in certain applications.

**Abstract:**

Textile-based wearable devices are rapidly gaining traction in the Internet of Things paradigm and offer distinct advantages for data collection and analysis across a wide variety of applications. Seamlessly integrating electronics in textiles remains a technical challenge, especially when the textiles’ essential properties, such as comfort, breathability, and flexibility, are meant to be preserved. This article investigates screen printing as a textile post-processing technique for electronic integration, and highlights its versatility, cost-effectiveness, and adaptability in terms of design and customization. The study examines two silver-based inks screen-printed on an Oxford polyester textile substrate with a focus on substrate preparation and treatment. Before printing, the textile samples were cleaned with nitrogen gas and then subjected to low-pressure oxygen plasma treatment. For comparative analysis, two samples printed on polyethylene terephthalate (PET) serve as a reference. The findings highlight the importance of plasma treatment in optimizing the printability of textiles and demonstrate that it notably improves the electrical properties of conductive inks. Despite some remaining challenges, the study indicates that screen-printed electronics show promising potential for advancing the development of e-textiles and sensor-integrated wearables.

## 1. Introduction

Early prototypes of “wearables” date back to the 1980s with Steve Mann’s research on “wearable computing” [1,2]. Since then, advancements in electronic miniaturization, enhanced computing capacities, and printed and flexible electronics have transformed the landscape of wearable technology. Now part of the Internet of Things, wearable technology is seamlessly integrated in our daily lives and makes it possible to collect and analyze data from the human body and its surrounding environment [3]. Common applications include, but are not limited to, well-being, fitness and healthcare [4,5,6,7,8], military [9], occupational health and safety [10], and artistic performances and exhibitions [11]. This article looks at textile-based wearables in particular. Examples of such products include smart garments [7], underwear [12], shoe insoles [13,14], gloves, and socks [15], which can be embedded with electrocardiograms, temperature and humidity sensors, heating elements, or other electronic elements.

While early-stage prototypes or proof-of-concept wearable devices can often be designed using copper wires, rigid components, and printed circuit boards, modern commercial wearable devices demand a higher level of integration. Integration must be imperceptible to users to ensure comfort while augmenting the functionality of garments with embedded electronics. This higher level of integration between textiles and electronics poses a significant challenge when it comes to developing and manufacturing wearable technology. According to the literature, this integration can be achieved in two primary ways: using conductive fibers and yarns to create electronic textiles, or using post-processing techniques, such as embroidery or printing, on textiles [16,17]. This study focuses on the latter approach, in particular, flatbed screen printing.

The choice to focus on flatbed screen printing was driven by the project’s alignment with the local context in Quebec (Canada). The apparel ecosystem in Quebec is predominantly comprised of small- and medium-sized enterprises (SMEs). While custom-made e-textiles that integrate conductive yarns may be cost-prohibitive for SMEs, screen printing offers a versatile and cost-effective alternative. In addition, the flatbed screen printing process is already well-established in the local apparel industry, so making use of it reduces the need for investment in machinery and training. Although screen printing shows strong potential to facilitate knowledge transfer from research facilities to production shop floors, challenges remain when it comes to printing high-quality conductive patterns on textile substrates due to their surface properties.

This study aims to address these challenges by evaluating the use of oxygen plasma treatment as a surface modification technique to enhance ink–textile contact and ink conductivity. The novelty of this work lies in its systematic comparison of two silver-based inks printed on a polyester textile substrate and its detailed analysis of how low-pressure oxygen plasma treatment affects ink–textile contact. By comparing textile samples with two polyethylene terephthalate (PET) reference samples, the study provides new insights into optimizing textile printability using treatment methods.

The remainder of this paper is structured as follows. Section 2 discusses the background literature on textile screen printing and substrate treatment. Section 3 introduces the materials and methods used in this study. Section 4 consolidates the results by combining the analysis of microscope images, thickness measurements, and electrical characterization. Section 5 presents the limitations and perspectives of the research, and Section 6 concludes this paper.

## 2. Background Literature

This section provides a concise overview of the literature pertaining to flatbed screen printing (Section 2.1); flexible substrates, including textiles, and potential treatment methods, such as encapsulation and plasma treatment (Section 2.2); and the application of silver-based inks on textiles (Section 2.3).

### 2.1. Flatbed Screen Printing

Flatbed screen printing is an additive manufacturing technique that is widely used to deposit functional materials on substrates, which makes it particularly valuable for the fabrication of wearable electronics [5,18,19]. This method utilizes a mesh screen to transfer ink onto a surface through a pattern of openings in order to create precise features in a highly controlled manner [20]. Screen printing is known for its simplicity, scalability, and cost-effectiveness, especially when compared to other printing methods like inkjet or gravure printing [8,17]. It can be used to deposit thick, highly viscous inks, which are essential for creating robust and functional traces. Moreover, it is compatible with various substrates, including plastics, textiles, ceramics, and metals, which makes it a versatile option that is suitable for a many different applications [21,22].

A primary benefit screen printing has over conventional coating or embedding techniques is its additive nature. Consequently, screen printing leads to a reduction in material waste, which in turn decreases production costs and environmental impact [20]. The fact that screen printing can be adapted to complex and irregular surfaces, such as those found in textiles, further enhances its appeal, particularly for wearable electronics [16]. As the demand for smart materials and intelligent textiles grows, screen printing is increasingly being considered a critical technology for the direct fabrication of electronic components on textiles because it makes it possible to create multifunctional garments that seamlessly integrate sensors, conductive paths, and other electronic elements [21]. Finally, its flexibility in terms of design and customization makes it an ideal solution for the apparel manufacturing industry.

### 2.2. Substrates and Treatment Methods

Screen printing on plastic substrates such as PET, polyimide, and thermoplastic polyurethane (TPU) has been extensively researched. Each substrate offers distinct advantages. PET, for instance, is known for its smooth surface, optical transparency, and suitability for high-resolution printing, which make it ideal for applications like flexible displays and sensors [21]. TPU, on the other hand, is favored for applications requiring stretchability and flexibility, such as wearable sensors and conformal devices.

Although plastic substrates provide a stable and uniform platform for screen printing, they commonly lack the comfort and breathability of textiles. This limits their use in wearable applications that require close contact with the human body. One solution that lies at the intersection of textiles and plastic substrates is to laminate a printed TPU film onto textiles. The TPU can either be laminated over the entire surface or laser-cut to form a patch limited to the printed area. While this is an interesting solution, lamination over the entire surface considerably limits breathability and comfort. Additionally, over time, delamination can occur between the TPU and textile, especially if the textile is stretchable. Consequently, research is exploring using textiles themselves as substrates. Since textiles are naturally flexible, soft, and breathable, they offer a more comfortable and functional alternative for wearable electronics.

Textiles can be categorized based on the structure of their yarns, which may be woven, non-woven, or knitted. At the microscopic level, yarns are composed of twisted or assembled fibers of varying diameters. The fibers used in textile production can be synthetic (e.g., nylon, polyester, acrylic) or organic (e.g., cotton, silk, wool) and assembled to produce textiles that are purely organic, fully synthetic, or a blend of the two. These parameters, which operate at different scales, impact the surface properties, including roughness, contact angle, porosity, and tension, all of which significantly affect printing resolution and ink absorption [23]. The ink’s degree of adherence to the textile and durability to wear also pose additional challenges.

To address the aforementioned challenges, several research studies have proposed to incorporate an encapsulation layer on top of the printed conductive ink [24,25,26]. This approach can be coupled with the printing of an interface layer on the textile beneath the ink [27,28]. The encapsulation layer improves the washability and durability of printed traces by protecting them [29]. It reduces cracking and delamination, and prevents an increase in relative resistance [26]. The interface layer, on the other hand, serves to smooth the surface and makes it possible to reduce the thickness of printed conductive inks and improve their mechanical properties [30]. However, encapsulation may not be suitable for certain sensor applications that require direct contact between the conductive ink and the environment, such as printed humidity sensors, touch sensors, or dry electrodes, as described in [24,31].

Alternatively, several studies have proposed to use plasma treatment as an effective surface modification method that can eliminate the need for additional material layers [16]. It involves exposing the substrate to ionized gas, which alters the substrate’s surface properties without affecting its bulk characteristics. Depending on the type of plasma used (e.g., argon, oxygen, or nitrogen), different effects can be achieved, including surface cleaning, activation, or functionalization [32,33,34]. When plasma treatment is applied to textiles, it can notably increase the surface energy and thereby enhance the wettability and hydrophilicity [32,35,36,37]. This improves the bond between the textile and the ink [33]. Plasma treatment can also be used to micro-etch the surface in order to create a rougher texture at the microscopic level [37] that further improves mechanical interlocking and adhesion between the printed ink and the textile fiber [19]. This enhanced adhesion can be particularly beneficial for applications requiring high durability, as it reduces the likelihood of ink delamination under mechanical stress, washing, or bending [19]. Finally, with respect to electrical properties, Deogaonkar’s findings indicate that plasma treatment enhances conductivity [37].

In this article, low-pressure oxygen plasma treatment is explored as it activates the surface, which enhances adhesion between the textile and the ink [33,38]. Oxygen plasma introduces oxygen-containing functional groups to the surface, which improves bonding with inks and enhances the overall performance and reliability of printing on textiles [34]. Regarding the duration of plasma treatment, Jelil pointed out that textiles require longer treatment time than films due to their larger surface area [33]. Several studies in the literature have explored plasma treatment on polyester substrates for periods ranging from 20 s to 5 min depending on the power output and the property improvements sought [39,40,41]. This study therefore utilizes 0-, 2-, and 4-min treatment durations, as reported in the Section 3.

### 2.3. Inks

Previous studies have printed silver-based inks on a wide range of substrates including plastics (e.g., PET, TPU), paper [20], and various types of textiles [21] measuring less than 1 mm in thickness. Silver-based inks have been successfully applied to a variety of knitted, woven, and non-woven textiles made from natural, synthetic, or blended fibers [24,25,27,42]. The versatility of silver-based inks extends to stretchable textiles, such as knitted fabrics [31] and spandex [26,28], which demonstrates that they are suitable for a wide variety of textile substrates. The conductive patterns printed on these textiles can tolerate repeat bending and stretching while maintaining their conductive properties, which makes them well-suited for wearable applications [19]. Finally, their excellent electrical conductivity further accounts for their widespread use in textile screen printing [24].

In the literature, carbon-based inks have also been explored on a variety of textile substrates ranging from cotton fabrics [43] to a cotton–polyester blend [44] and non-woven viscose [19]. In this study, silver-based inks are preferred as they have superior electrical properties.

## 3. Materials and Methods

The materials and study parameters that were utilized to print and characterize the samples are presented below.

### 3.1. Screen Printing Process

This study investigates conductive structures that were screen-printed in three orientations relative to the squeegee motion: vertically (parallel), horizontally (perpendicular), and diagonally (at a 45-degree angle). Each pattern consists of lines having a fixed length of 20 mm and widths ranging from 0.25 mm to 1 mm to evaluate print resolution, line fidelity, and electrical performance. The structures were oriented different ways to determine how the printing direction affects ink deposition, edge definition, and conductivity, which are key parameters when it comes to optimizing the screen printing of fine conductive features in electronic textile applications.

Screen printing was conducted using a P-200S flatbed screen printer from Keko Equipment, Žužemberk, Slovenija. Mesh with a count of 90 T (90 threads per centimeter or 230 threads per inch) was coated with a 1 mil (25.4 μm) thick layer of photo-sensitive emulsion. This emulsion acts as a stencil by blocking the flow of ink in all areas except where the design is exposed and developed so the ink passes through only the intended pattern. A 120 mm long squeegee set at a 30° slope was used to uniformly spread the ink across the screen at a speed of 90 mm/s [28]. Single-layer deposition was performed, and the printed image size (10 × 10 cm) was limited by the frame dimensions (12 × 12 cm).

### 3.2. Substrate and Treatment

Two substrates were considered for this study. The first, a 125 μm untreated PET film, MELINEX® ST506 5 mil, Mylar®, Chester, VA, USA was used as a reference substrate for comparative analysis. The second, an uncoated Oxford polyester textile referred to as “Montana 2009” from Tonitex, Montréal, QC, Canada, was selected on the basis of its minimal stretchability and low porosity (290 gsm/600 dens), which are characteristics that were identified as preferable by Kim et al. [24].

In order to modify the surface chemistry of the polyester textile, a two-step treatment process is employed. First, the surface was cleaned with nitrogen gas, which helps to remove loosely bound contaminants and moisture without introducing reactive oxygen species and thereby preserves the integrity of the substrate while promoting better adhesion during subsequent plasma treatment. Then, oxygen plasma treatment was applied for either 2 or 4 min using Diener Pico Version 6 equipment, Ebhausen, Germany. The oxygen flow rate was maintained at 20 mL/min during plasma treatment, and the power was set to 145 W. The distance between the top of the vacuum chamber and the substrate in was approximately 15 cm. Since plasma treatment is subject to aging effect [33,36], all samples were treated and printed on the same day to preserve the treatment’s effectiveness and minimize aging effect. Plasma treatment was performed in the morning and was followed by screen printing approximately two hours later, in the afternoon.

### 3.3. Selected Inks

Two silver-based inks that are suitable for flatbed screen printing and textile substrates were selected for their high conductivity, stretchability, and flexibility. The inks are SINK 127-07 from Creative Materials, Ayer, MA, USA and SInk01NP from Nano Paint, São Cosme, Portugal. Both inks have been used in recent studies. SINK 127-07—hereinafter “Ink 1”—was screen-printed on a TPU film and was selected for its stretchability [45], while SInk01NP—hereinafter “Ink 2”—was used in two research projects [46,47] for its stretchability, high conductivity, and compatibility with screen printing and textile substrates. Their properties are summarized in Table 1. Note that a higher viscosity is required for printing on textiles, preferably in the range of 16,000–24,000 cPs [48].

The curing conditions were selected based on the thermal properties of the textile substrate to ensure a temperature below its melting point. These properties were identified using differential scanning calorimetry with a PerkinElmer instrument, Springfield, IL, USA.

### 3.4. Characterization Methods

The morphologies of the printed samples, which differed in their use of Ink 1 or Ink 2 and their textile treatment conditions, were studied using a VHX-7000 digital microscope, Mississauga, ON, Canada from Keyence with 20× magnification. In addition, a Hitachi TM3000 scanning electron microscope (SEM), Tokyo, Japan with 200× magnification and an accelerating voltage of 10 kV was used to explore surface transformations under the different plasma treatment conditions and observe ink penetration within the textile substrates. The SEM was also employed to measure the thickness of the printed layers, as the significant surface roughness of the textile substrates posed challenges for accurate measurement with a profilometer. Appendix B discusses the choice to use a razor blade with mechanical support over ion milling and laser cutting for sample preparation. The thickness of the layer printed on the two PET reference samples was measured using a Tencor^®^ P-17 profilometer from KLA, Milpitas, CA, USA.

The electrical properties of the printed lines were evaluated using a four-point probe system from Ossila, Sheffield, United Kingdom in ambient conditions (relative humidity = 25 ± 3%; relative temperature = 20.5 ± 2 °C), which made it possible to comprehensively understand how morphological variations affect electrical performance.

## 4. Results

The Section 4 starts with an analysis of the printed samples and the images obtained using the digital microscope (Section 4.1). Section 4.2 presents the thickness measurements obtained from cross-sectional images. Section 4.3 focuses on electrical characterization.

### 4.1. Microscope Analysis

**Figure 1:**
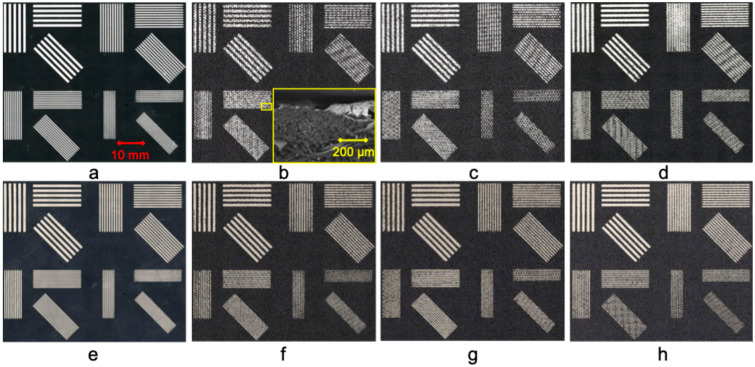
Microscopy images of the screen-printed samples, all captured at the same scale for consistent comparison. The first row (**a**–**d**) features samples printed with Ink 1, and the second row (**e**–**h**) features those printed with Ink 2. Images (**a**,**e**) are the samples printed on the PET substrate for reference. The textile treatment variations are as follows: (**b**,**f**) no treatment, serving as control samples; (**c**,**g**) nitrogen cleaning followed by a 2 min oxygen plasma treatment; (**d**,**h**) nitrogen cleaning and an extended 4 min oxygen plasma treatment. The inset of (**b**) presents a cross-sectional SEM image showing the distribution of ink across the textile yarns. For a more detailed view, refer to Figure A1 and Figure A2 in Appendix A. Adapted from [49].

Figure 1 presents a detailed view of the screen-printed samples, with all images shown at the same scale for consistent comparison. Each ink was also applied to an untreated PET substrate as a reference to ensure optimal screen–printer alignment and support comparison.

The first row (a–d) of Figure 1 features samples printed with Ink 1, and the second row (e–h) showcases those printed with Ink 2. The images highlight significant ink dispersion on the untreated samples (b,f), which show the textile yarns beneath and indicate poor ink–textile contact. The images demonstrate that plasma treatment enhances the ink–substrate interaction, particularly images (d) and (h), which have the highest observed print quality due to the treatment parameters used. These two samples exhibit precise ink coverage that conceals the textile yarns and ensures a uniform printed surface. Notably, printing lines that are narrower than the yarn diameter—in this case, 0.506 mm—leads to increased printing complexities and a higher incidence of discontinuities in the lines. This observation is supported by the SEM image of the surface that is inset in Figure 1b. It shows that the ink follows the yarns of the textile.

### 4.2. Thickness Measurements

**Figure 2:**
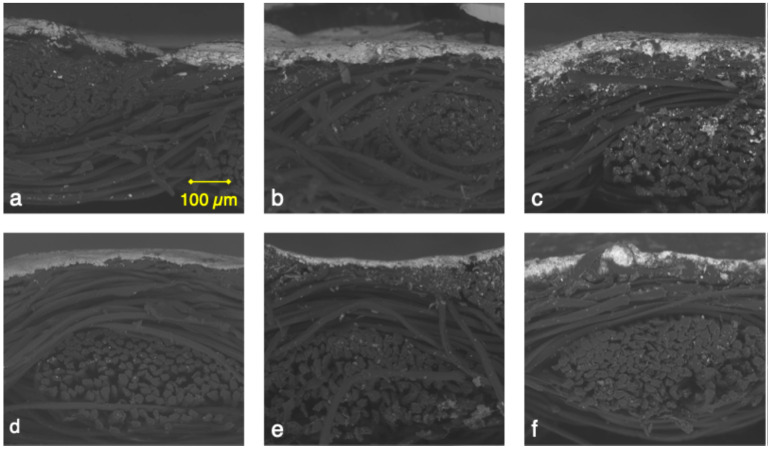
SEM cross-section images of the screen-printed samples, all captured at 200× magnification and the same scale for consistent comparison. Images (**a**–**c**) are of samples printed with Ink 1, and (**d**–**f**) are samples printed with Ink 2. The textile treatment variations are as follows: (**a**,**d**) no treatment, serving as control samples; (**b**,**e**) nitrogen cleaning followed by a 2 min oxygen plasma treatment; (**c**,**f**) nitrogen cleaning followed by an extended 4 min oxygen plasma treatment.

SEM observation was employed to determine the thickness of the printed lines. Figure 2 presents cross-sectional images of samples printed with Ink 1 (a–c) and Ink 2 (d–f). In the untreated textile samples (a,d), the ink primarily remains on the surface of the fabric, with minimal penetration into the textile structure. However, the plasma-treated samples, especially (c) and (f), display enhanced ink infiltration between the textile yarns, which improves overall ink–textile contact. This increased penetration is likely due to the plasma treatment enhancing surface wettability, which enables the ink to better conform to the structure of the yarns.

Table 2, row 2 provides the average thickness measurements determined from the SEM images based on five measurements taken for each condition. The samples printed with Ink 1 exhibit greater thickness, which might be attributable to Ink 1 having higher silver solid content than Ink 2 (see Table 1). This difference in thickness influences the mechanical stability of the printed structures and therefore makes the choice of ink and substrate treatment critical to optimize performance in electronic textile applications.

### 4.3. Electrical Characterization

The average sheet resistance values of the fabricated samples, which are detailed in Table 2, row 3, provide quantitative evidence that reinforces the qualitative observations made via optical microscopy. Each value was again based on five measurements taken of the same printed pattern. The PET samples exhibit the lowest resistance, which is attributable to the substrate’s inherent surface properties. Moreover, the measurements reveal a systematic reduction in sheet resistance with the progression from no treatment to nitrogen cleaning plus 2 min of oxygen plasma treatment and then to nitrogen cleaning plus 4 min of oxygen plasma treatment. This trend is consistent across the samples printed with Ink 1 and Ink 2 with slight variations in the absolute resistance values. These findings confirm that plasma treatment significantly enhanced the electrical conductivity of the printed lines by improving ink–textile contact. The improved print quality and uniformity observed in the optical microscopy and SEM images also support this.

## 5. Discussion and Research Perspectives

This study demonstrates the role plasma treatment plays in optimizing the electrical properties of printed silver-based inks, as evidenced by Table 2. In what follows, different points related to the literature are discussed in line with the results.

First, the reduction in sheet resistance with increased plasma treatment time can be explained by the fact the extended treatment more effectively removes surface contaminants and roughens the textile substrate, which increases the contact area between the ink and the substrate [33,37]. This in turn leads to more continuous conductive paths, which is in line with the optical microscopy observations. As for the samples’ electrical properties, those results echo Deogaonkar’s findings [37], with an improvement in conductivity following plasma treatment.

Second, in line with the observations formulated in Section 4.1, it is worth mentioning that various parameters including ink composition, ink viscosity, and surface tension can impact visual print quality and are discussed in the literature [8,50]. To mitigate process variability, training and testing was conducted prior to printing the samples analyzed.

Visual observations were complemented with cross-sectional analyses that made it possible to highlight that ink penetration improved with plasma treatment. Ink penetration, like print quality, can be impacted by ink properties and other printing parameters [19,25,42]. It increased for both Ink 1 and Ink 2 with 4 min of plasma treatment as shown in Figure 2c,f. This higher ink penetration can positively impact print durability [51]. Print durability is discussed in the next paragraph with a focus on research perspectives.

One major challenge associated with printed textile electronics is maintaining good electrical properties throughout the wearable device life cycle. Printed textile electronics must withstand bending, stretching, twisting, and abrasion to remain durable during wear [52]. In some applications, wearable devices may need to be washed, which tests their mechanical, chemical, and heat resistance [26]. The absence of washability tests and extensive mechanical tests in this study can limit the range of applications this study’s findings can be considered for. Additionally, how sweat affects ink and ink ageing require further investigation [19]. To envision a broader range of applications, the ability to repeatedly stretch beyond 100% is an additional challenge that should be studied.

Future studies will address these limitations by focusing on synthetic stretch textiles and highly stretchable silver-based inks. Also, samples will undergo wash tests, cyclic stretching, and bending tests to compare their performance. Along with these tests, peel or tape testing will be included in the protocol to evaluate mechanical durability. In the event of inconclusive results, encapsulation using a biodegradable material will be considered. Indeed, the environmental impact of wearables should not be overlooked [7,20,53,54]. Preference will therefore be given to inks and processes with less environmental impact in future works.

## 6. Conclusions

This paper explores screen printing in the context of wearable devices with the application of two different silver-based inks on an Oxford polyester textile combined with varying durations of oxygen plasma treatment, and how these parameters influence printing outcomes. The analysis not only underscores the critical role surface treatment plays in optimizing the electrical properties of printed e-textiles, but also highlights the intricate interplay between print resolution, substrate characteristics, and treatment parameters that must be considered to achieve the desired electrical performance. Notably, the compatibility of silver-based ink with plasma-treated textile surfaces improves ink transfer and ink–textile contact, and further optimizes the printing process. The results observed during this study can inform future optimization when fabricating conductive patterns for e-textile applications requiring both high print fidelity and low electrical resistance.

To conclude, this preliminary study lays the foundation for future research in the domain of electronic textiles and wearable devices. In terms of applications, examples include printing dry electrodes [31] and reusable humidity sensors for urine detection in underwear [55,56], as well as facilitating their integration by printing conductive tracks between the printed sensors and electronic circuits. The perspectives of printable electronics on textiles are vast for sensing applications.

## Figures and Tables

**Table 1:** Properties of the silver-based inks used.

Properties	Inks	
	Ink 1 SINK 127-07 Creative Materials	Ink 2 SInk01NP Nano Paint
Viscosity, cPs	18,000–25,000	8000–20,000
Solid content, %	84	65
Surface resistivity, Ω/sq	0.008	0.029
Curing conditions	60 min at 70 °C	

**Table 2:** Comparison of the average thickness and average sheet resistance of samples printed with conductive inks.

		PET	No Treatment	N_2_ Cleaning + O_2_ Plasma 2 min	N_2_ Cleaning + O_2_ Plasma 4 min
Thickness (μm)	Ink 1	21.50 ± 0.04	26.54 ± 2.61	26.68 ± 3.81	29.99 ± 5.43
	Ink 2	16.75 ± 0.06	15.78 ± 1.78	17.08 ± 1.83	21.86 ± 2.03
Sheet resistance (Ω/sq)	Ink 1	4.56 ± 0.00	14.34 ± 0.30	13.83 ± 0.21	11.70 ± 0.13
	Ink 2	7.79 ± 0.00	16.57 ± 0.34	15.95 ± 0.19	14.68 ± 0.15

**Figure A1:**
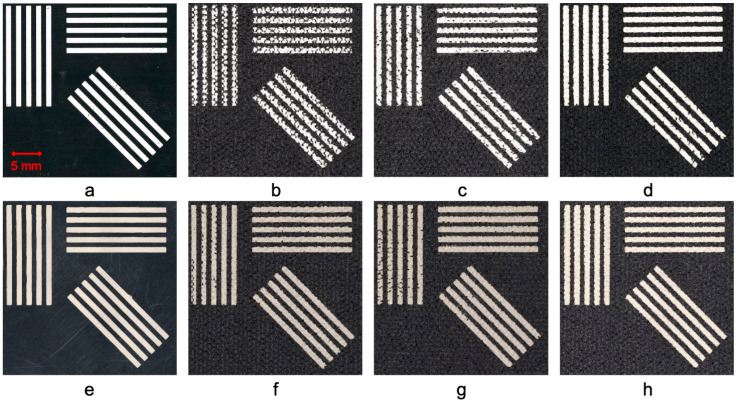
Zoomed-in microscopy images of the screen-printed samples, focusing on the lines with a length of 20 mm and a width of 1 mm in the upper left corner of the images shown in Figure 1. All images were captured at the same scale for consistent comparison. The first row (**a**–**d**) features samples printed with Ink 1, and the second row (**e**–**h**) features those printed with Ink 2. Images (**a**,**e**) are the samples printed on the PET substrate for reference. The textile treatment variations are as follows: (**b**,**f**) no treatment, serving as control samples; (**c**,**g**) nitrogen cleaning followed by a 2 min oxygen plasma treatment; (**d**,**h**) nitrogen cleaning and an extended 4 min oxygen plasma treatment.

**Figure A2:**
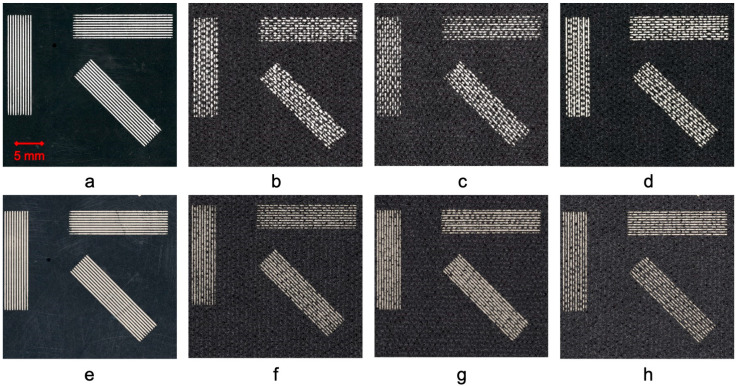
Zoomed-in microscopy images of the screen-printed samples, focusing on the lines with a length of 20 mm and a width of 0.25 mm in the upper left corner of the images shown in Figure 1. All images were captured at the same scale for consistent comparison. The first row (**a**–**d**) features samples printed with Ink 1, and the second row (**e**–**h**) features those printed with Ink 2. Images (**a**,**e**) are the samples printed on the PET substrate for reference. The textile treatment variations are as follows: (**b**,**f**) no treatment, serving as control samples; (**c**,**g**) nitrogen cleaning followed by a 2 min oxygen plasma treatment; (**d**,**h**) nitrogen cleaning and an extended 4 min oxygen plasma treatment.

**Figure A3:**
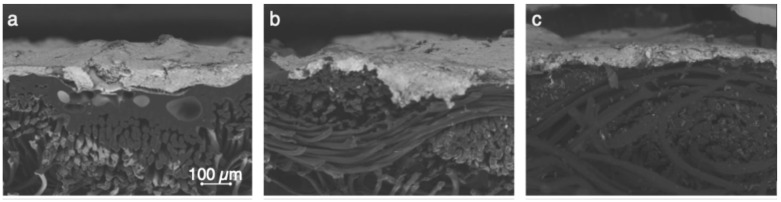
SEM cross-section images of the screen-printed samples cut using different techniques: (**a**) laser cutting; (**b**) ion milling; (**c**) razor blade cutting. All images were captured at the same scale for consistent comparison.

## Data Availability

The original contributions presented in this study are included in the article. Further inquiries can be directed to the corresponding author.

## Abbreviations

The following abbreviations are used in this manuscript:

PET	Polyethylene terephthalate
SEM	Scanning electron microscope
SMEs	Small- and medium-sized enterprises
TPU	Thermoplastic polyurethane

## Appendix A

To provide more detailed views of the printed structures, Figure A1 presents zoomed-in microscopy images of the samples shown in Figure 1. These magnified images highlight the line edges, surface coverage, and overall print quality, and offer additional visual support for the comparison of different treatment conditions.

## Appendix B

Determining the average thickness of conductive traces screen-printed on an Oxford polyester textile presents several challenges. The highly uneven surface profile of the textile samples makes the use of profilometers impractical, as these devices require a relatively smooth and uniform surface to provide accurate measurements. The rough and flexible nature of textiles interferes with the stylus’s ability to maintain consistent contact and results in unreliable data. One alternative is to conduct cross-sectional observations using a scanning electron microscope (SEM)—in this case, a Hitachi TM3000, Tokyo, Japan—which makes it possible to measure the trace thickness directly. However, the conductive inks used were highly flexible and malleable when cured, similar to the textile substrate. This flexibility meant that any attempt to cut a sample in half often resulted in deformation, which complicated the observation and measurement process.

These challenges were addressed by benchmarking various cutting methods to obtain cross-sections of the printed traces for thickness measurement. The methods evaluated include laser cutting, ion milling, and razor blade cutting with support.

Laser cutting uses a laser to make a perpendicular incision through the textile and ink layers. However, due to the low melting point of the polyester substrate used, the laser caused the edges of the sample to melt, as seen in Figure A3a, which complicated the analysis.

Ion milling removes material by bombarding the surface with accelerated ions that erode the targeted area until the material is fully cut through. Although it is effective, Figure A3b shows that some areas beneath the ink layer were partially melted, which indicates incomplete removal of the material. Additionally, this method has a long processing time.

Razor blade cutting with support involves positioning the textile sample between fixed metallic supports and using a razor blade to make a perpendicular incision across the printed trace. This method minimizes deformation in the perpendicular direction to the ink layer and preserves the integrity of the thickness measurement as illustrated in Figure A3c. Moreover, it avoids contamination, which makes it the preferred cutting technique for accurate cross-section analysis.
